# Endophytic *Burkholderia* sp. SSG as a potential biofertilizer promoting boxwood growth

**DOI:** 10.7717/peerj.9547

**Published:** 2020-07-16

**Authors:** Ping Kong, Chuanxue Hong

**Affiliations:** Hampton Roads Agricultural Research and Extension Center, Virginia Polytechnic Institute and State University (Virginia Tech), Virginia Beach, VA, United States of America

**Keywords:** Boxwood endophyte, *Burkholderia* sp. SSG, IAA and siderophore production, Nitrogen fixation, Phosphate solubilization, Plant growth promotion, Biofertilizer, Biocontrol agent

## Abstract

**Background:**

*Burkholderia* sp. SSG is a bacterial endophyte isolated from boxwood leaves showing a resistant response to infection by the boxwood blight pathogen *Calonectria pseudonaviculata*. SSG acted as a protective and curative biocontrol agent for boxwood blight and as a bio-sanitizer of disease inoculum in the field. Many gene clusters involved in antibiotic production and plant growth promotion (PGP) were found in the genome, giving this endophyte great application potential as a treatment for plant protection. However, the PGP features have not been documented. This study investigated the plant growth promotion activity of SSG in boxwood.

**Methods:**

To determine whether SSG is a plant growth promoting bacterium, four PGP traits, auxin and siderophore production, nitrogen fixation and phosphate solubilization, were examined in the laboratory with colorimetric or agar plate assays. The plant growth promoting activity of SSG was tested on three boxwood varieties characterized by slow, intermediate and fast growth rates, namely Justin Brouwers, Buddy and Winter Gem, respectively. These plants were drenched with an SSG cell suspension or water and washed plant weight was compared before and after treatment to determine growth changes after 10 months.

**Results:**

The SSG culture was sustainable on nitrogen free media, suggesting that SSG may fix atmospheric nitrogen. It was also a strong phosphate solubilizer and a potent siderophore and indole-3-acetic acid (IAA) producer. Significant growth promotion was observed on boxwood cultivars Justin Brouwers, Buddy and Winter Gem 10 months after plant roots were drenched with SSG cells. The growth rate of treated plants was 76.1, 58.3, and 37.3% higher than that of the control, respectively. The degree of growth promotion was significantly different among plant varieties, notably more pronounced with the slow and intermediate growers. This study demonstrates that the SSG bacterium has multiple PGP traits and is a prospective plant biofertilizer.

## Introduction

Endophytes have recently received considerable attention because of their ability to promote plant growth and suppress plant pathogens (Díaz Herrera et al., 2016; Eljounaidi, Lee & Bae, 2016; Joy & Parke, 1994; Nejad & Johnson, 2000; Reinhold-Hurek & Hurek, 2011; Santoyo et al., 2016). *Burkholderia* sp. SSG was isolated from boxwood leaves showing a resistant response to infection by *Calonectria pseudonaviculata* (*Cps*): the initial water-soaked lesions which developed 48 h after inoculation with *Cps* disappeared with no subsequent disease development (Kong & Hong, 2020b). As an environmental member of the *Burkholderia cepacia* complex (Bcc) , SSG differs from the clinical strains involved in lung infections of immunocompromised patients (Vandamme et al., 1997) by the onion maceration test response, RecA restriction fragment length polymorphism and lack of the Burkholderia cepacia Epidemic Strain Marker (BCESM) (Kong & Hong, 2020b). Recent genome sequencing (Kong & Hong, 2020a) has confirmed that SSG does not have the cable pini subunit gene (*cbl* A) for BCESM (Mahenthiralingam et al., 2000; Mahenthiralingam, Simpson & Speert, 1997; Sajjan et al., 1995). It also reveals the absence of several multiloci that are used for Bcc typing (Baldwin et al., 2005). More interestingly, the SSG genome contains genes encoding traits that are uncommon in Bcc clinical strains, such as those involved in nitrogen fixation and production of bacteriocin (Bevivino et al., 1994; Gonzalez & Vidaver, 1979). These traits indicate a low human health risk and high potential of SSG as a biocontrol agent for plant diseases and biofertilizer for plant production.

Boxwood blight is a deadly disease of boxwood caused by *Cps* (Daughtrey, 2019; LeBlanc, Salgado-Salazar & Crouch, 2018). Leaves inoculated with the pathogen can develop blight symptoms within 72 h (Kong & Hong, 2018). SSG provided nearly complete protection from the disease when used as a foliar treatment on boxwood plants before or shortly after plant infection by *Cps* (Kong & Hong, 2020b). Such protection is superior to any biocontrol product or other potential biocontrol agents evaluated to date (Kong, 2019; Kong & Hong, 2017; Kong & Hong, 2019; Yang & Hong, 2018; Yang & Hong, 2017). When used to treat diseased leaf debris in the field, SSG diminished production of inocula and mitigated disease development (Kong & Hong, 2020b).

Biocontrol agents for plant diseases are often plant growth promoters (Compant et al., 2005; Pal & McSpadden Gardener, 2006). This is particularly true for Bcc environmental strains (Batista et al., 2018; Bevivino et al., 1998; Germida & Walley, 1996; Ghosh et al., 2016; Sopheareth et al., 2013; Trân Van et al., 2000). Many of these Bcc strains were reported to have a high capacity for antibiotic production (Depoorter et al., 2016), as well as production of other metabolites that can promote plant growth through phosphate solubilization, ethylene regulation with 1-aminocyclopropane -1-carboxylate (ACC) deaminase and sequestering iron (Batista et al., 2018; Ghosh et al., 2016; Santoyo et al., 2016; Santoyo, Orozco-Mosqueda & Govindappa, 2012; Trân Van et al., 2000). Whole genome sequencing of SSG indicated its greater capacity than other members of the environmental Bcc for antibiotic synthesis and production of other secondary metabolites beneficial for plant growth (Kong & Hong, 2020a). However, SSG has not been verified as a plant growth promoting (PGP) bacterium. This study aims to explore the potential of SSG as a biofertilizer. Four PGP traits: nitrogen fixation, phosphate solubilization and production of IAA (Indole-3-Acetic Acid) and siderophores were examined through colorimetric or agar plate assays. SSG was also evaluated for plant growth promotion on three boxwood varieties through drench application.

## Materials & Methods

### SSG Culture growth conditions

*Burkholderia* sp. SSG, from the Virginia Tech Collection of Phytophthora and Beneficial Microbes (VTC) of the World Data Center for Microorganism (WDCM1197), was grown and maintained on potato dextrose agar (PDA), nutrient agar (NA) or in nutrient broth (NB) (Becton, Dickinson and Company, Spark, MO, USA) at 25−28 °C. For a fresh culture, a streak plate was prepared from the stored culture and incubated for 48 h.

### IAA production

IAA production by SSG was determined quantitatively using the colorimetric method (Liaqat & Eltem, 2016) with a minor modification. Specifically, 4 ml of NB containing 4 mg tryptophan was inoculated with a single colony from a 48-h SSG fresh culture plate. After a 72-h incubation at 28 °C, 1.5 ml of SSG broth culture or the control, NB without SSG, was centrifuged at 13,523 g for five minutes. 0.5 ml of the supernatant was then mixed with 1 ml Salkowski’s reagent in a 1.5-ml tube and incubated at 23 °C for 30 min. The reaction with SSG supernatant was then measured for absorbance at 530 nm after blanking with the control on a DU800® spectrophotometer (Beckman Coulter, Indianapolis, IN, USA). The assay was run in triplicate and repeated once. A standard curve constructed with an IAA dilution series (Sigma-Aldrich, St. Louis, MO, USA) at a range of 0.1–300 µg ml/l was used for quantification of IAA in the sample.

### Nitrogen fixation ability

Nitrogen fixation was determined by growing SSG on nitrogen-free agar medium as described previously (Liaqat & Eltem, 2016). Specifically, nitrogen-free agar plates were streaked with fresh SSG colonies from a PDA culture. Nutrient agar plates were used as a positive control. Plates were incubated at 25 °C for 4 days and examined for bacterial growth. The assay was conducted in triplicate and repeated once.

### Phosphate solubilization

The ability of SSG to solubilize phosphate was determined using the National Botanical Research Institute’s Phosphate (NBRIP) broth or agar medium and the colorimetric method (Nautiyal, 1999; Pradhan & Raj Pokhrel, 2013) with minor modifications. For the plate assay, three sterilized Whatman filter paper disks were placed on NBRIP agar plates at the points of an equilateral triangle. A 10-µl aliquot of SSG cell culture stock was pipetted onto each disk. Control disks received the same amount of nutrient broth without SSG. All plates were incubated at 27 °C for seven days, then examined for development of a halo around the disks. For the broth colorimetric assay, 150 mg Ca_3_(PO_4_)_2_ as an insoluble form of phosphate was added to 30 ml NBRIP broth, to which 0.3 ml of an overnight (16–18 h) SSG culture in NB or NB alone (the control) was added. After incubation on a shaker at 27 °C for seven days, the culture was centrifuged at 13,416 g for 10 min. The supernatant was autoclaved for 20 min and stored at 4 °C. To determine soluble phosphate release into the solution, 1 ml of the supernatant or its dilution was added to 2 ml of 2.5% ammonium molybdate and 0.5 ml of 10 mol/l sulfuric acid, mixed with 1 ml of 0.5 mol/l hydrazine hydrate solution, then brought to 25 ml with SDW. The NB control was used as a blank and the SSG culture supernatant was measured for absorbance at 840 nm on a DU800® spectrophotometer. When the absorbance of a sample was one or smaller, soluble phosphate was calculated by sample absorbance/0.1235 + 0.0018. When the absorbance of a sample was one or greater, soluble phosphate was calculated after a 100× dilution (Pradhan & Raj Pokhrel, 2013). Both assays included three replicates and were repeated once.

### Siderophore production

Siderophore production by SSG was determined using blue agar medium containing chrome azurol S (CAS) and the indicator hexadecyltrimethylammonium bromide (Schwyn & Neilands, 1987). Specifically, the media plates were streaked with SSG and incubated at 25 °C. Plate color change was examined after 48 h. Plates with a color change from blue to yellow were recorded as positive. This assay included three replicate plates and the assay was repeated twice.

### Plant treatment and growth measurement

Three boxwood cultivars with different growth rates, *Buxus sempervirens* ‘Justin Brouwers’ (slow), ‘Buddy’ (intermediate) and *B. microphylla var. japonica* ‘Winter Gem’ (fast), were used in this study. Two plants were grown in 3.8-liter containers and maintained in a greenhouse before use. One week before SSG treatment in November 2018, plants were separated and rinsed with tap water to remove potting mix. Cleaned individual plants were weighed after drying with a paper towel, then repotted in a mixture of Scotts® Premium Potting Soil (Marysville, OH) and pine bark (Pacific Mulch Inc, Henderson, NC) at 1: 2 in 3.8-liter containers. These plants were watered manually to saturate the soil, followed by drip irrigation every other day for one min.

Plants were treated by drenching with an SSG cell suspension prepared by inoculating 3 flasks each containing 150 ml NB with 1 ml from a 5ml overnight broth culture. After incubation at 28 °C on a shaker for 40 h, each culture was pooled and centrifuged at 8,275 g for 15 min. The cell pellets were resuspended in 500 ml dH_2_O after supernatant was removed. For treatment, a 50-ml aliquot of SSG resuspension at 10^8^ cfu/ml or the same volume of water without SSG was evenly poured onto the potting mix around plants in containers. After treatment, containers were arranged in a randomized complete block design and drip irrigation was resumed after two days. In March 2019 plants were moved out of the greenhouse to a gravel pad with overhead irrigation. In September 2019 plants were removed from containers, washed free of soil mix and weighed as in November 2018. Plant growth was measured by the difference in plant weight between the beginning and end of the experiments. The experiment was conducted three times with an interval of a week.

### Statistical analysis

Plant growth data from three repeated experiments were subjected to a homogeneity test and subsequently pooled for further analyses. Analysis of variance was conducted using the Statistical Analysis Software Version 9.4 (SAS Institute, Cary, NC). Treatment means were separated by boxwood cultivar according to the least significance difference at *P* = 0.05.

## Results

### Plant growth promotion traits of SSG

**Figure 1:**
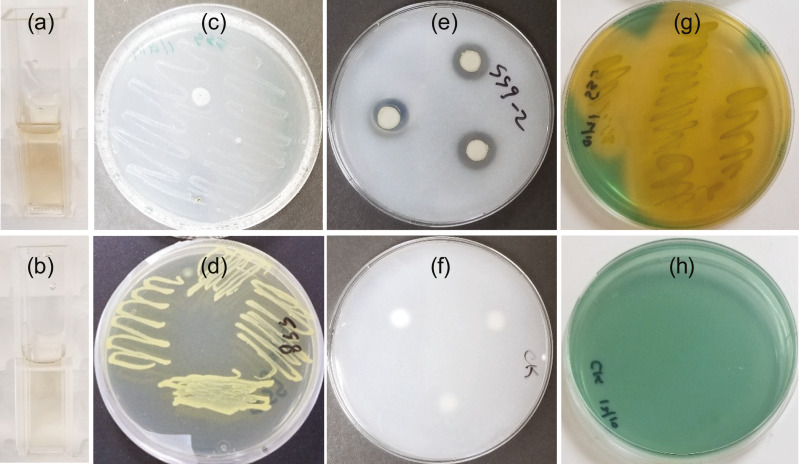
SSG plant growth promoting traits as shown in a colorimetric or plate assay. (A) Light pink color produced at 2 days showing IAA production; (C) Growth on nitrogen free media at 4 days showing nitrogen fixation; (E) Halo produced around disks at 7 days showing phosphate solubilization and (G) Yellow color change at 3 days showing siderophore production. (B), (D), (F) and (H) are images of the control tube or plate for A, C, E, and G, respectively.

IAA was detected in the cell free supernatant two days after NB broth containing tryptophan was inoculated with SSG cells (Fig. 1A). The estimated yield was 2.9–4.5 µg/ml. The amount of IAA detected did not change with longer growth periods, suggesting limited use of tryptophan. No color change occurred in the control (Fig. 1B).

SSG grew on nitrogen-free medium (Fig. 1C) although not as well as on nitrogen-rich medium, NB (Fig. 1D).

Phosphate solubilization by SSG was confirmed by both plating and colorimetric methods. A clear halo developed around the SSG disks on NBRIP agar medium within three days. These halos enlarged with increasing incubation time. They were 14 mm (±0.3) in diameter by the 7th day (Fig. 1E). No halos formed on any of the control plates (Fig. 1F). The solubilized phosphate measured colorimetrically after 7 days was 206.4 ppm (±5.0), approximately 21% of the insoluble form of phosphate.

The blue agar chrome azurol S assay detected siderophore production by SSG. The agar turned yellow 48 h after the plate was streaked with SSG (Fig. 1G) and no color change occurred on the NB streaked control (Fig. 1H).

### Effect of SSG on boxwood plant growth

**Figure 2:**
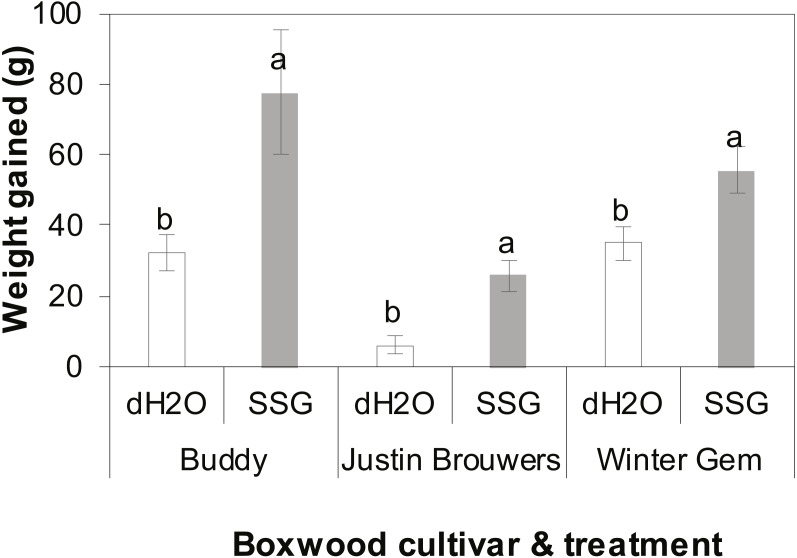
Boxwood plant growth of three cultivars—Buddy (intermediate), Justin Brouwers (slow) and Winter Gem (fast) as affected by SSG cell suspension (SSG) or control (dH_2_O) drench over a 10-month period. Each column is a mean of nine replicate plants from three repeated experiments. Standard error bars are presented on top of the columns. Columns within each cultivar topped with different letters differed according to the least significant difference at *P* = 0.05.

The growth rate of three boxwood varieties was measured 10 months after drenching the container mix with an SSG cell suspension or water. There was no difference between three repeated experiments (*P* = 0.6905) nor interaction between cultivar and treatment (*P* = 0.2121), cultivar and experiment (*P* = 0.1366) and between treatment and experiment (*P* = 0.2434). However, there was significant difference between treatments with and without SSG and the difference varied with cultivar (*P* < 0.0001). SSG consistently promoted plant growth of all three boxwood cultivars when compared to the control (Fig. 2). Specifically, the growth increase in SSG treated plants was 58%, 76% and 37% greater than that of the controls in Buddy (*P* = 0.0236), Justin Brouwers (*P* = 0.0014), and Winter Gem (*P* = 0.0190), respectively.

## Discussion

This study investigated the plant growth promotion activity of SSG on boxwood. Although SSG was isolated from leaves, it stimulated plant growth when applied as a root treatment. When compared to the nontreated controls, 76% greater growth rate was observed in the SSG-treated plants of slower growing ‘Justin Brouwers’, a cultivar used in a previous study evaluating disease suppression by SSG (Kong & Hong, 2020b). In that study, an increase in leaf number was observed when SSG culture was used to treat diseased leaf debris added to containers with healthy plants. However, since boxwood blight incidence also decreased with the treatment, it was not certain whether the leaf increase was a result of normal plant growth after disease reduction. This study confirms the plant growth promotion ability of SSG and suggests that the increase in leaf number observed previously may be attributed to the treatment. The current study revealed a trend that slower growing cultivars ‘Justin Brouwers’ and ‘Buddy’ benefited more from SSG treatment than the fast-growing cultivar ‘Winter Gem’. All three showed a significant increase in growth after SSG treatment compared to their controls. It is not clear why SSG was more effective on the slow and intermediate than the fast-growing cultivar; one possibility is that the effect of SSG may be overruled by other genetic factors in the faster growing cultivar which may be less dependent on environmental conditions for growth. SSG has been shown to be able to survive in soil and rhizosphere (Kong & Hong, 2020b). However, how it behaves in the rhizosphere and how it responds to plant genetic factors remain to be further studied.

SSG is a plant growth promoting bacterium. IAA is the basic and most potent auxin natively occurring and functioning in plants and it regulates leaf and flower development (Benková et al., 2003; Ludwig-Müller, 2011). IAA was detected in SSG cell free culture supernatant. To our knowledge, SSG is the first leaf endophytic burkholderial bacterium producing IAA, as other IAA-producing *Burkholderia* are found in the stem, root and rhizosphere (Mendes et al., 2007; Weilharter et al., 2011). IAA production by SSG was relatively low, 2.9–4.5 µg/ml, compared to some non-*Burkholderia* bacterial endophytes that produce 9.6–43 µg/ml (Liaqat & Eltem, 2016). However, it is not clear whether such yield is common in IAA producing *Burkholderia* due to lack of quantitative data. Interestingly, genes encoding tryptophan-2-monooxygeanse or tryptophan transaminase were not found in the SSG genome (Kong & Hong, 2020a). These enzymes play important roles in the pathways of tryptophan-dependent IAA biosynthesis in bacteria (*Pseudomonas* and *Agrobacterium*) and plants (Zhao, 2010; Zhao, 2012). It is not understood how IAA was produced without these genes, although there are genes for tryptophan production. Whether SSG may use a different pathway for IAA production is still a question to be answered.

Another distinctive trait of SSG is nitrogen fixation as indicated by SSG growth on nitrogen-free medium. Nitrogen fixation has been found in various endophytic bacteria (Estrada-De Los Santos, Bustillos-Cristales & Caballero-Mellado, 2001; Ghosh et al., 2016; Liaqat & Eltem, 2016; Trân Van et al., 2000), but it is uncommon for Bcc (Gonzalez & Vidaver, 1979). SSG is the second member of Bcc that can fix nitrogen, following *B. vietnamiensis* (Gillis et al., 1995). This ability of SSG corresponds well with its genome compacity for the trait. Many genes involved in nitrogen fixation and regulation have been found in the SSG genome (Kong & Hong, 2020a). These genes include the nitrogenase gene (eg. *NifQ*) (Hoffman et al., 2014), the *hglE* cluster, heterocyst glycolipid synthase-like PKS involving nitrogen fixation in cyanobacteria heterocyst (Campbell, Cohen & Meeks, 1997; Fan et al., 2005), and genes for nitrogen fixation and regulation such as *pstN* and *glnB* (Fan et al., 2005; Michiels et al., 1998). With this capacity, SSG can modulate nitrogen acquisition and metabolism.

Treatment of seed or soil with phosphate-solubilizing bacteria can improve crop yield by releasing insoluble and fixed forms of phosphorus such as rock phosphate (Khan, Zaidi & Wani, 2007; Qureshi et al., 2012; Reijnders, 2014). Weak phosphate-solubilizing bacteria do not produce a halo in the plate assay (Nautiyal, 1999). The halo formed by SSG suggests that this bacterium is a potent phosphate solubilizer. The amount produced as quantified with the colorimetric method (Pradhan & Raj Pokhrel, 2013) is similar to that reported for some strong phosphate solubilizing bacterial endophytes including *Burkholderia* spp. (Ghosh et al., 2016; Liaqat & Eltem, 2016; Qureshi et al., 2012). Optical density of the supernatant of phosphate-solubilizing bacterial culture in NBRIP with Ca_3_(PO_4_)_2_ has been used to measure soluble form of phosphorus in other studies (Ghosh et al., 2016; Liaqat & Eltem, 2016). However, since there are no comparative studies on these methods, values of soluble form of phosphorus by these bacteria from different research may not be comparable.

Siderophores from microorganisms can be used by a plant for iron nutrition, soil heavy metal stress alleviation and plant pathogen suppression (Glick, 2012). SSG was a potent siderophore producer as shown by the plating method. This is consistent with the data from SSG genome sequencing revealing more than 100 genes involved in siderophore biosynthesis, assembly and metabolism (Kong & Hong, 2020a). However, it is not clear whether SSG may be different from other plant growth promoting Bcc in terms of siderophore composition and number due to limited research on plant growth promoting Bcc species.

## Conclusions

This study confirms that the potent biocontrol agent, boxwood endophytic *Burkholderia* sp. SSG, is also a plant growth promoter. Plant growth increased by 37–76% when the bacterium was applied as a drench to containerized boxwood. Four important plant growth promoting traits predicted by SSG genome sequencing were also verified in the laboratory. IAA production, nitrogen fixation, phosphorus solubilization and siderophore production were confirmed in this endophyte. These traits demonstrate its potential as a biofertilizer. To elucidate *Burkholderia* sp. SSG as a potent biofertilizer, future studies should include more genomic prospection of the bacterium, such as acquisition, transfer and metabolism of the growth hormone, nitrogen, phosphorus and iron, as well as protein secretion systems, especially the Type VI Secretion Systems that are widespread in *Burkholderia* spp. and very powerful to suppress bacterial or eukaryotic cells. To promote application of SSG in crop production and health, assessment of its biocontrol spectrum for plant pathogens and development of effective formulations are warranted.

##  Supplemental Information

10.7717/peerj.9547/supp-1 Supplemental Information 1 Raw measurements of boxwood plant weight before and after treatment

10.7717/peerj.9547/supp-2 Supplemental Information 2 Statistical analysis for Fig. 2

## Additional Information and Declarations

### Data Availability

The following information was supplied regarding data availability: The raw measurements and statistics for Fig. 2 are available in the Supplemental Files.

## References

[ref-1] Baldwin A, Mahenthiralingam E, Thickett KM, Honeybourne D, Maiden MCJ, Govan JR, Speert DP, LiPuma JJ, Vandamme P, Dowson CG (2005). Multilocus sequence typing scheme that provides both species and strain differentiation for the *Burkholderia cepacia* complex. Journal of Clinical Microbiology.

[ref-2] Batista BD, Lacava PT, Ferrari A, Teixeira-Silva NS, Bonatelli ML, Tsui S, Mondin M, Kitajima EW, Pereira JO, Azevedo JL, Quecine MC (2018). Screening of tropically derived, multi-trait plant growth- promoting rhizobacteria and evaluation of corn and soybean colonization ability. Microbiological Research.

[ref-3] Benková E, Michniewicz M, Sauer M, Teichmann T, Seifertová D, Jürgens G, Friml J (2003). Local, efflux-dependent auxin gradients as a common module for plant organ formation. Cell.

[ref-4] Bevivino A, Sarrocco S, Dalmastri C, Tabacchioni S, Cantale C, Chiarini L (1998). Characterization of a free-living maize-rhizosphere population of *Burkholderia cepacia*: effect of seed treatment on disease suppression and growth promotion of maize. FEMS Microbiology Ecology.

[ref-5] Bevivino A, Tabacchioni S, Chiarini L, Carusi MV, Del Gallo M, Visca P (1994). Phenotypic comparison between rhizosphere and clinical isolates of *Burkholderia cepacia*. Microbiology.

[ref-6] Campbell E, Cohen M, Meeks JC (1997). A polyketide-synthase-like gene is involved in the synthesis of heterocyst glycolipids in *Nostoc punctiforme* strain ATCC 29133. Archives of Microbiology.

[ref-7] Compant S, Duffy B, Nowak J, Clément C, Barka EA (2005). Use of plant growth-promoting bacteria for biocontrol of plant diseases: principles, mechanisms of action, and future prospects. Applied and Environmental Microbiology.

[ref-8] Díaz Herrera S, Grossi C, Zawoznik M, Groppa MD (2016). Wheat seeds harbour bacterial endophytes with potential as plant growth promoters and biocontrol agents of *Fusarium graminearum*. Microbiological Research.

[ref-9] Daughtrey ML (2019). Boxwood blight: threat to ornamentals. Annual Review of Phytopathology.

[ref-10] Depoorter E, Bull MJ, Peeters C, Coenye T, Vandamme P, Mahenthiralingam E (2016). *Burkholderia*: an update on taxonomy and biotechnological potential as antibiotic producers. Applied Microbiology and Biotechnology.

[ref-11] Eljounaidi K, Lee SK, Bae H (2016). Bacterial endophytes as potential biocontrol agents of vascular wilt diseases—review and future prospects. Biological Control.

[ref-12] Estrada-De Los Santos P, Bustillos-Cristales RO, Caballero-Mellado J (2001). *Burkholderia*, a genus rich in plant-associated nitrogen fixers with wide environmental and geographic distribution. Applied and Environmental Microbiology.

[ref-13] Fan Q, Huang G, Lechno-Yossef S, Wolk CP, Kaneko T, Tabata S (2005). Clustered genes required for synthesis and deposition of envelope glycolipids in *Anabaena* sp. strain PCC 7120. Molecular Microbiology.

[ref-14] Germida JJ, Walley FL (1996). Plant growth-promoting rhizobacteria alter rooting patterns and arbuscular mycorrhizal fungi colonization of field-grown spring wheat. Biology and Fertility of Soils.

[ref-15] Ghosh R, Barman S, Mukherjee R, Mandal NC (2016). Role of phosphate solubilizing *Burkholderia* spp. for successful colonization and growth promotion of *Lycopodium cernuum* L. (*Lycopodiaceae*) in lateritic belt of Birbhum district of West Bengal, India. Microbiological Research.

[ref-16] Gillis M, Van Van T, Bardin R, Goor M, Hebbar P, Willems A, Segers P, Kersters K, Heulin T, Fernandez MP (1995). Polyphasic taxonomy in the genus *Burkholderia* leading to an emended description of the genus and proposition of *Burkholderia* Vietnamiensis sp. Nov. for N2-fixing isolates from rice in Vietnam. International Journal of Systematic and Evolutionary Microbiology.

[ref-17] Glick BR (2012). Plant growth-promoting bacteria: Mechanisms and applications. Scientifica.

[ref-18] Gonzalez CF, Vidaver AK (1979). Bacteriocin, plasmid and pectolytic diversity in *Pseudomonas cepacia* of clinical and plant origin. Microbiology.

[ref-19] Hoffman BM, Lukoyanov D, Yang Z-Y, Dean DR, Seefeldt LC (2014). Mechanism of nitrogen fixation by nitrogenase: the next stage. Chemical Reviews.

[ref-20] Joy AE, Parke JL, Bailey WG, Whitehead C, Proctor JTA, Kyle JT (1994). Biocontrol of *Alternaria* leaf blight on American ginseng by *Burkholderia cepacia* AMMD.

[ref-21] Khan MS, Zaidi A, Wani PA (2007). Role of phosphate-solubilizing microorganisms in sustainable agriculture—a review. Agronomy for Sustainable Development.

[ref-22] Kong P (2019). Evaluation of a novel endophytic *Pseudomonas lactis* strain for control of boxwood blight. Journal of Environmental Horticulture.

[ref-23] Kong P, Hong C (2017). Biocontrol of boxwood blight by *Trichoderma* koningiopsis Mb2. Crop Protection.

[ref-24] Kong P, Hong C (2018). Host responses and impact on the boxwood blight pathogen, *Calonectria pseudonaviculata*. Planta.

[ref-25] Kong P, Hong CX (2019). Utilization of plant endophytes for control of boxwood blight. https://www.sna.org/resources/Documents/19resprosec06.pdf.

[ref-26] Kong P, Hong C (2020a). Complete genome sequence of a boxwood endophyte *Burkholderia* sp. SSG with broad biotechnological application potential. Biotechnology Reports.

[ref-27] Kong P, Hong C (2020b). A potent *Burkholderia* endophyte against boxwood blight caused by *Calonectria pseudonaviculata*. Microorganisms.

[ref-28] LeBlanc N, Salgado-Salazar C, Crouch JA (2018). Boxwood blight: an ongoing threat to ornamental and native boxwood. Applied Microbiology and Biotechnology.

[ref-29] Liaqat F, Eltem R (2016). Identification and characterization of endophytic bacteria isolated from *in vitro* cultures of peach and pear rootstocks. 3 Biotech.

[ref-30] Ludwig-Müller J (2011). Auxin conjugates: their role for plant development and in the evolution of land plants. Journal of Experimental Botany.

[ref-31] Mahenthiralingam E, Bischof J, Byrne SK, Radomski C, Davies JE, Av-Gay Y, Vandamme P (2000). DNA-based diagnostic approaches for identification of *Burkholderia cepacia* complex, *Burkholderia vietnamiensis, Burkholderia multivorans, Burkholderia stabilis, Burkholderia cepacia* genomovars I and III. Journal of Clinical Microbiology.

[ref-32] Mahenthiralingam E, Simpson DA, Speert DP (1997). Identification and characterization of a novel DNA marker associated with epidemic *Burkholderia cepacia* strains recovered from patients with cystic fibrosis. Journal of Clinical Microbiology.

[ref-33] Mendes R, Pizzirani-Kleiner AA, Araujo WL, Raaijmakers JM (2007). Diversity of cultivated endophytic bacteria from sugarcane: genetic and biochemical characterization of *Burkholderia cepacia* complex isolates. Applied and Environmental Microbiology.

[ref-34] Michiels J, Van Soom T, D’Hooghe I, Dombrecht B, Benhassine T, De Wilde P, Vanderleyden J (1998). The Rhizobium etli rpoN locus: DNA sequence analysis and phenotypical characterization of rpoN, ptsN, and ptsA mutants. Journal of Bacteriology.

[ref-35] Nautiyal CS (1999). An efficient microbiological growth medium for screening phosphate solubilizing microorganisms. FEMS Microbiology Letters.

[ref-36] Nejad P, Johnson PA (2000). Endophytic bacteria induce growth promotion and wilt disease suppression in oilseed rape and tomato. Biological Control.

[ref-37] Pal KK, McSpadden Gardener B (2006). Biological control of plant pathogens. The plant health instructor. American Phytopathological Society.

[ref-38] Pradhan S, Raj Pokhrel M (2013). Spectrophotometric determination of phosphate in sugarcane juice, fertilizer, detergent and water samples by molybdenum blue method. Scientific World.

[ref-39] Qureshi MA, Ahmad ZA, Akhtar N, Iqbal A, Mujeeb F, Shakir MA (2012). Role of phosphate solubilizing bacteria (PSB) in enhancing P availability and promoting cotton growth. Journal of Animal and Plant Sciences.

[ref-40] Reijnders L (2014). Phosphorus resources, their depletion and conservation, a review. Resources, Conservation and Recycling.

[ref-41] Reinhold-Hurek B, Hurek T (2011). Living inside plants: bacterial endophytes. Current Opinion in Plant Biology.

[ref-42] Sajjan US, Sun L, Goldstein R, Forstner JF (1995). Cable (cbl) type II pili of cystic fibrosis-associated *Burkholderia* (*Pseudomonas*) cepacia: nucleotide sequence of the cblA major subunit pilin gene and novel morphology of the assembled appendage fibers. Journal of Bacteriology.

[ref-43] Santoyo G, Moreno-Hagelsieb G, Del Carmen Orozco-Mosqueda M, Glick BR (2016). Plant growth-promoting bacterial endophytes. Microbiological Research.

[ref-44] Santoyo G, Orozco-Mosqueda MD, Govindappa M (2012). Mechanisms of biocontrol and plant growth-promoting activity in soil bacterial species of *Bacillus* and *Pseudomonas*: a review. Biocontrol Science and Technology.

[ref-45] Schwyn B, Neilands JB (1987). Universal chemical assay for the detection and determination of siderophores. Analytical Biochemistry.

[ref-46] Sopheareth M, Chan S, Naing KW, Lee YS, Hyun HN, Kim YC, Kim KY (2013). Biocontrol of late blight (*Phytophthora capsici*) disease and growth promotion of pepper by *Burkholderia cepacia* MPC-7. The Plant Pathology Journal.

[ref-47] Trân Van V, Berge O, Ngô Kê S, Balandreau J, Heulin T (2000). Repeated beneficial effects of rice inoculation with a strain of *Burkholderia vietnamiensison* early and late yield components in low fertility sulphate acid soils of Vietnam. Plant and Soil.

[ref-48] Vandamme P, Holmes B, Vancanneyt M, Coenye T, Hoste B, Coopman R, Revets H, Lauwers S, Gillis M, Kersters K, Govan JRW (1997). Occurrence of multiple genomovars of *Burkholderia cepacia* in cystic fibrosis patients and proposal of *Burkholderia multivorans* sp. nov. International Journal of Systematic and Evolutionary Microbiology.

[ref-49] Weilharter A, Mitter B, Shin MV, Chain PSG, Nowak J, Sessitsch A (2011). Complete genome sequence of the plant growth-promoting endophyte *Burkholderia phytofirmans* strain PsJN. Journal of Bacteriology.

[ref-50] Yang X, Hong CX (2017). Evaluation of biofungicides for control of boxwood blight on boxwood. Plant Disease Management Reports.

[ref-51] Yang X, Hong C (2018). Biological control of boxwood blight by *Pseudomonas protegens* recovered from recycling irrigation systems. Biological Control.

[ref-52] Zhao Y (2010). Auxin biosynthesis and its role in plant development. Annual Review of Plant Biology.

[ref-53] Zhao Y (2012). Auxin biosynthesis: a simple two-step pathway converts tryptophan to indole-3-acetic acid in plants. Molecular Plant.

