# Supplementary material for: Endophytic Burkholderia sp. SSG as a potential biofertilizer promoting boxwood growth
Source: PeerJ. 2020 Jul 16;8:e9547. doi: 10.7717/peerj.9547 (PMC7369026; doi:10.7717/peerj.9547)
Supplement: Supplemental Information 2 [file peerj-08-9547-s002.pdf]

The ANOVA Procedure

Class Level Information		
Class	Levels	Values
cv	3	BD JB WG
treat	2	dH2O SSG
exp	3	I II III

Number of Observations Read	54
Number of Observations Used	54

Dependent Variables: w (g/mon)

Source	DF	Sum of Squares	Mean Square	F Value	Pr > F
Model	13	344. 1074282	26. 4698022	4. 49	0. 0001
Error	40	235. 5597737	5. 8889943		
Corrected Total	53	579. 6672019			

R-Square	Coeff Var	Root MSE	w Mean
0. 593629	62. 52775	2. 426725	3. 881037

Source	DF	Anova SS	Mean Square	F Value	Pr > F
cv	2	149. 3295676	74. 6647838	12. 68	< .0001
treat	1	110. 3445345	110. 3445345	18. 74	< .0001
exp	2	4. 4026089	2. 2013045	0. 37	0.6905
cv*treat	2	18. 9892196	9. 4946098	1. 61	0.2121
cv*exp	4	43. 7954224	10. 9488556	1. 86	0.1366
treat*exp	2	17. 2460751	8. 6230376	1. 46	0.2434

The SAS System

The ANOVA Procedure

cv=BD

Class Level Information		
Class	Levels	Values
treat	2	dH2O SSG

Number of Observations Read	18
Number of Observations Used	18

Dependent Variables: w (g/mon)

Source	DF	Sum of Squares	Mean Square	F Value	Pr > F
Model	1	92. 5389427	92. 5389427	6. 26	0. 0236
Error	16	236. 6016442	14. 7876028		
Corrected Total	17	329. 1405869			

R-Square	Coeff Var	Root MSE	w Mean
0. 281153	69. 77870	3. 845465	5. 510944

Source	DF	Anova SS	Mean Square	F Value	Pr > F
treat	1	92. 53894272	92. 53894272	6. 26	0. 0236

t Tests (LSD) for w

Note: This test controls the Type I comparisonwise error rate, not the experimentwise error rate.

Alpha	0.05
Error Degrees of Freedom	16
Error Mean Square	14.7876
Critical Value of t	2. 11991
Least Significant Difference	3. 8429

Means with the same letter
are not significantly different.

t Grouping	Mean	N	treat
A	7. 778	9	SSG
B	3. 244	9	dH2O

The SAS System

The ANOVA Procedure

cv=JB

Class Level Information		
Class	Levels	Values
treat	2	dH2O SSG

Number of Observations Read	18
Number of Observations Used	18

Dependent Variable: w(g/mon)

Source	DF	Sum of Squares	Mean Square	F Value	Pr > F
Model	1	17. 29896200	17. 29896200	14. 92	0. 0014
Error	16	18. 54956778	1. 15934799		
Corrected Total	17	35. 84852978			

R-Square	Coeff Var	Root MSE	w Mean
0. 482557	67. 38455	1. 076730	1. 597889

Source	DF	Anova SS	Mean Square	F Value	Pr > F
treat	1	17. 29896200	17. 29896200	14. 92	0. 0014

t Tests (LSD) for w

Note: This test controls the Type I comparisonwise error rate, not the experimentwise error rate.

Alpha	0.05
Error Degrees of Freedom	16
Error Mean Square	1.159348
Critical Value of t	2. 11991
Least Significant Difference	1.076

Means with the same letter
are not significantly different.

t Grouping	Mean	N	treat
A	2. 5782	9	SSG
B	0. 6176	9	dH2O

The SAS System

The ANOVA Procedure

cv=WG

Class Level Information		
Class	Levels	Values
treat	2	dH2O SSG

Number of Observations Read	18
Number of Observations Used	18

Dependent Variable: w(g/mon)

Source	DF	Sum of Squares	Mean Square	F Value	Pr > F
Model	1	19. 49584939	19. 49584939	6. 80	0. 0190
Error	16	45. 85266822	2. 86579176		
Corrected Total	17	65. 34851761			

R-Square	Coeff Var	Root MSE	w Mean
0. 298337	37. 33483	1. 692865	4. 534278

Source	DF	Anova SS	Mean Square	F Value	Pr > F
treat	1	19. 49584939	19. 49584939	6. 80	0. 0190

t Tests (LSD) for w

Note: This test controls the Type I comparisonwise error rate, not the experimentwise error rate.

Alpha	0. 05
Error Degrees of Freedom	16
Error Mean Square	2. 865792
Critical Value of t	2. 11991
Least Significant Difference	1. 6917

Means with the same letter
are not significantly different.

t Grouping	Mean	N	treat
A	5.5750	9	SSG
B	3.4936	9	dH2O

The SAS System

w (g/mon)

Obs	cv	treat	mW	seW
1	BD	dH2O	3.24356	0.51128
2	BD	SSG	7.77833	1.73918
3	JB	dH2O	0.61756	0.25449
4	JB	SSG	2.57822	0.43917
5	WG	dH2O	3.49356	0.46230
6	WG	SSG	5.57500	0.65048
